# Critical air pollutant assessments and health effects attributed to PM_2.5_ during and after COVID-19 lockdowns in Iran: application of AirQ^+^ models

**DOI:** 10.3389/fpubh.2023.1120694

**Published:** 2023-05-25

**Authors:** Safiye Ghobakhloo, Amir Hossein Khoshakhlagh, Gholam Reza Mostafaii, Kai-Jen Chuang, Agnieszka Gruszecka-Kosowska, Pariya Hosseinnia

**Affiliations:** ^1^Department of Environmental Health Engineering, School of Health, Kashan University of Medical Sciences, Kashan, Iran; ^2^Department of Occupational Health Engineering, School of Health, Kashan University of Medical Sciences, Kashan, Iran; ^3^School of Public Health, College of Public Health, Taipei Medical University, Taipei, Taiwan; ^4^Faculty of Geology, Geophysics, and Environmental Protection, Department of Environmental Protection, AGH University of Science and Technology, Krakow, Poland; ^5^Department of Public Health, Garmsar Branch, Islamic Azad University, Garmsar, Iran

**Keywords:** air pollution, COVID-19, air quality index (AQI), AirQ+ modeling, lockdown

## Abstract

**Objectives:**

The aim of this study was to evaluate changes in air quality index (AQI) values before, during, and after lockdown, as well as to evaluate the number of hospitalizations due to respiratory and cardiovascular diseases attributed to atmospheric PM_2.5_ pollution in Semnan, Iran in the period from 2019 to 2021 during the COVID-19 pandemic.

**Methods:**

Daily air quality records were obtained from the global air quality index project and the US Environmental Protection Administration (EPA). In this research, the AirQ+ model was used to quantify health consequences attributed to particulate matter with an aerodynamic diameter of <2.5 μm (PM_2.5_).

**Results:**

The results of this study showed positive correlations between air pollution levels and reductions in pollutant levels during and after the lockdown. PM_2.5_ was the critical pollutant for most days of the year, as its AQI was the highest among the four investigated pollutants on most days. Mortality rates from chronic obstructive pulmonary disease (COPD) attributed to PM_2.5_ in 2019–2021 were 25.18% in 2019, 22.55% in 2020, and 22.12% in 2021. Mortality rates and hospital admissions due to cardiovascular and respiratory diseases decreased during the lockdown. The results showed a significant decrease in the percentage of days with unhealthy air quality in short-term lockdowns in Semnan, Iran with moderate air pollution. Natural mortality (due to all-natural causes) and other mortalities related to COPD, ischemic heart disease (IHD), lung cancer (LC), and stroke attributed to PM_2.5_ in 2019–2021 decreased.

**Conclusion:**

Our results support the general finding that anthropogenic activities cause significant health threats, which were paradoxically revealed during a global health crisis/challenge.

## Introduction

Air pollution has become one of the main problems in developing countries (1, 2). One of the consequences of air pollution is its harmful and destructive effects on human health, which cause a decrease in life expectancy and a reduction in the environmental benefits of work (3). Air pollution has short- and long-term adverse effects depending on the concentrations of chemical substances. On the contrary, the duration of exposure by living organisms is also one of the most critical factors in the susceptibility to pollution (4). The effects on human health led to increased treatment costs and work pressure on doctors, and in the long run, it could cause fatigue and burnout in medical staff. Therefore, the damage caused by this phenomenon is very significant from both human cultural and economic aspects. It should be noted that human health has been the most critical factor of researchers' attention (5). Moreover, the International Agency for Research on Cancer (IARC) has categorized outdoor air pollution as a group 1 human carcinogen (6). Also, according to studies by the WHO, 4.6 million deaths are attributed annually to diseases related to air pollution (7). Scientific research conducted in the last two decades has shown that particles are one of the critical pollutants from the point of view of health effects (such as eye irritation, dry throat, runny nose, sneezing, coughing, tiredness, irritability, difficulty concentrating, headaches, cardiovascular effects, hypertension, obesity, and type 2 diabetes mellitus), cancer health risks, and mortality (8). The WHO estimated the annual cost of air pollution in Austria, France, and Switzerland to be about 30 billion pounds and that air pollution-related deaths accounted for 6% of all deaths in those countries (9). Particulate matter (PM) with an aerodynamic diameter of ≤2.5 μm (PM_2.5_) significantly affects health and increases mortality rates due to respiratory, cardiovascular, and lung diseases (10). Long-term exposure to PM causes a 6% increase in mortality for each 10 μg m^−3^ increase in its concentration in the air (11). An increase of 10 μg m^−3^ in PM_2.5_ was reported to result in a 14% increase in lung cancer and a 12% increase in vascular diseases (12). At the end of 2019, the COVID-19 virus was first reported in Wuhan, China, and after a short time, it spread worldwide. The pandemic caused by COVID-19 has had direct impacts on changes to air quality index (AQI) values (13). Long-term exposure to PM is related to the occurrence of diseases such as high blood pressure, diabetes, and cardiovascular diseases (14). Research into the long-term effects showed that the mortality index caused by COVID-19 had higher values in areas with higher air PM concentrations (15). Mortality rates in those areas were higher due to underlying diseases (16). Also, in Iran, the death rate caused by contracting COVID-19 had a direct relationship with suspended particles in the air, and thus cities with higher pollution also had higher death rates over time (17). The reason for this could be underlying diseases caused by long-term pollution. Also, due to traffic restrictions and reduced factory activities, concentrations of the majority of air pollutants decreased in most parts of the world compared to the recent months before the spread of COVID-19 (18). Further research in this field assessed the impacts of the spread of COVID-19 on changes in air pollution quality and environmental health in Iran (19, 20). One study highlighted the impact of the severity of the coronavirus disease on the decrease in air pollution caused by carbon dioxide in the world's metropolises (21). The spread of COVID-19 in Iran since February 2020 prompted the closure of many businesses and reduce travel for people in the community to reduce the spread of the disease. In Iran, the first official case of COVID-19 transmission was detected on February 19, 2020, in Qom (22), and shortly thereafter it spread throughout the entire country (23). To control the global spread of COVID-19, the government issued a state of emergency, which included “lockdown” restrictions on the movement of people and on transportation and a ban on economic, educational, sports, cultural, and religious activities (24). Several studies have investigated the relationship between lockdown and AQI changes and the effect of pollutants on the number of COVID-19 cases and mortality (Table 1). A positive association between AQI and daily confirmed cases of COVID-19 was observed in China (27). Based on the evidence obtained from the USA for the determination of air pollution exposure and COVID-19 death rate, there was an association between an increase of only 1 μg m^−3^ in PM_2.5_ and 95% CI with an 8% increase in COVID-19 mortality rate (28). In 107 Italian territorial areas, 1 μg m^−3^ increase in PM_2.5_ 9% in the average Covid-19 mortality rate (29). The PM_2.5_, PM_10_, NO_2_, and O_3_, emissions have an increase of 2.24, 1.76, 6.94, and 4.76% in the daily numbers of confirmed COVID-19 patients in China, respectively (32). In England, a significant association has been reported between air quality (NO_2_, O_3_, PM_2.5_, and PM_10_) and mortality associated with COVID-19 infection was observed (34). Since air pollution is related to emissions from combustion in factories and for heating purposes, as well as from the transportation sector, it was expected that pollutants would simultaneously decrease during the appearance of COVID-19 waves with “lockdown” measures. The objective of the study was to analyze historical data on air pollution from 2019 to 2021 in Semnan, Iran with regard to the appearance of COVID-19 waves and lockdown episodes to define potential improvements in air quality. The detailed aims of the study were to (1) evaluate changes in AQI values in Semnan, Iran in three analyzed periods: before lockdown (BF: 1 March 2019 to 27 February 2019), during lockdown (LD: 1 March 2020 to 27 February 2020), and after lockdown (AF: 1 March 2021 to 27 February 2021); (2) determine the critical/dominant pollutant in AQI for each research period, and (3) quantify and estimate health effects attributed to PM_2.5_ using the AirQ^+^ model in Semnan in the mentioned periods. As it was expected that the research results would indicate significant impacts of reducing anthropogenic emissions to reduce atmospheric pollution in Iran, this research results might become useful for relevant authorities for implementing strategies for air quality protection.

**Table 1:** Air pollution effect research on the number of COVID-19 cases and mortality.

**Pollutants**	**Country**	**Impact**
Air quality index (AQI)	10 polluted cities in the world (25)	The concentration of air pollutants has decreased in all world cities during the lockdown period.
Air quality index (AQI)	China, Japan, and India (26)	In Wuhan and Mumbai, the percentage of unhealthy days decreased significantly during quarantine and continued after quarantine. PM_2.5_ was a critical pollutant for all cities.
Air quality index (AQI)	China (27)	A direct association was observed between AQI and COVID-19-confirmed patients.
PM_2.5_	United States (28)	1 μg/m^3^ increase in PM_2.5_ led to 8% increase in COVID-19 death rate
PM_2.5_	Italy (107 Italian territorial areas) (29)	1 μg/m^3^ increase in PM_2.5_ 9% in the average COVID-19 mortality rate
PM_10_	Italy(North) (30)	The daily limit value of PM_10_ has significantly increased the number of COVID-19 cases.
PM_10_, NO_2_, SO_2_, and CO	United States (California) (31)	All the pollutants had a significant correlation with the COVID-19 epidemic.
PM_2.5_, PM_10_, NO_2_, O_3_	China (120 cities) (32)	10 μg/m^3^ increase in NO_2_, PM_10_, CO, SO_2_, and O_3_ due to 2.24%, 1.76%, 6.94%, 7.79 %, and 4.76% increase in the daily number of confirmed patients, respectively.
PM_2.5_, PM_10_, O_3_, NO_2_, SO_2_, CO	Korea (33)	Significant correlations were observed between COVID-19 incidence in South Korea and NO_2_, SO_2_, and CO.
NO_2_, O_3_, PM_2.5_, PM_10_	England (34)	A significant association has been reported between air quality and mortality associated with COVID-19 infection.

## Materials and methods

### Site description

**Figure 1:**
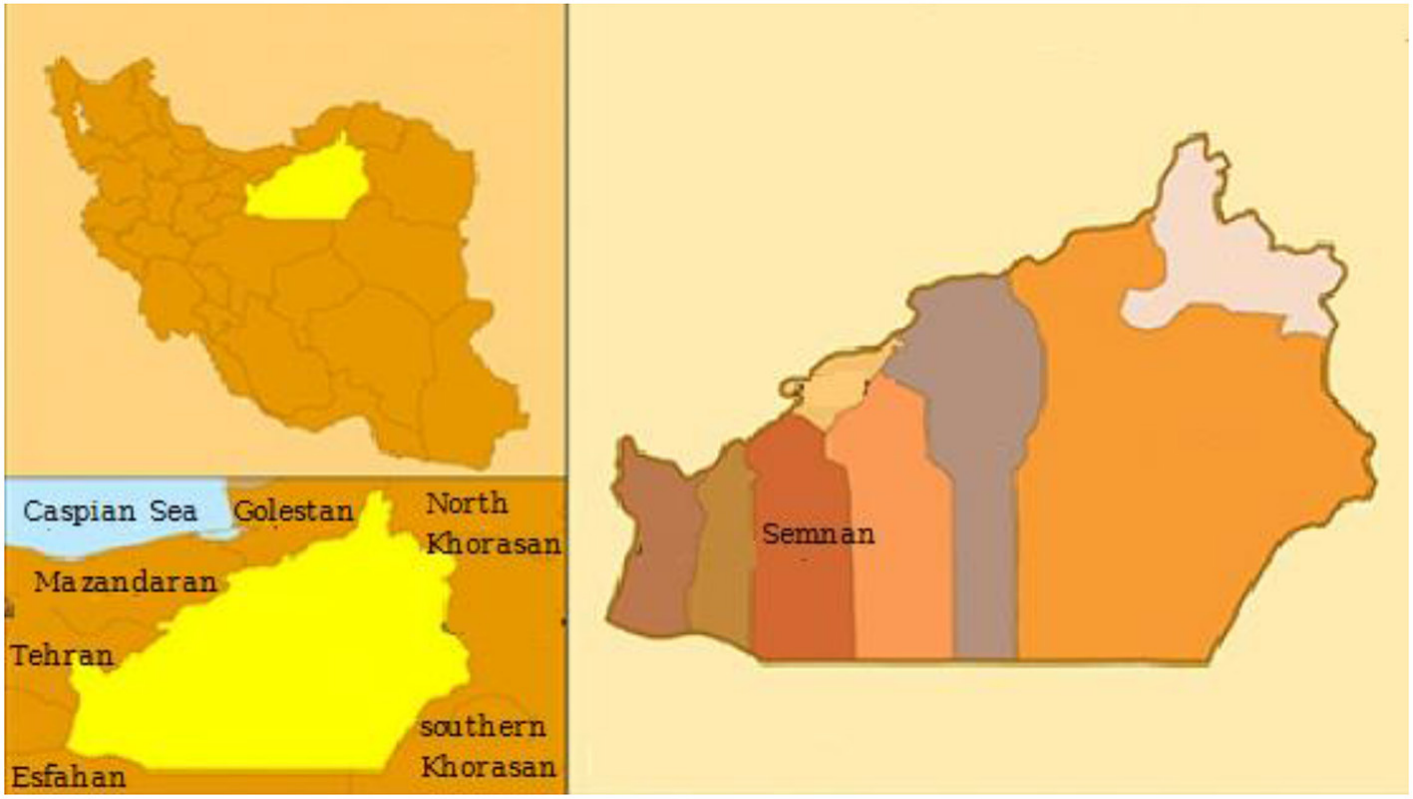
Study area.

Descriptive analytical research was conducted by a cross-sectional study in the city of Semnan, Iran (35°58′N, 53°43'E) located in north-central Iran and in the eastern part of Tehran Province, with a population of 224,145 adults (Figure 1). According to the recent research, 600,000 ha of Semnan Province, Iran is in the center of a wind erosion crisis, and 137,000 ha are affected by wind erosion. Concentrations of the pollutants, NO_2_, CO, PM_2.5_, and O_3_, investigated in this research were obtained from an air quality monitoring station located on the northern side of Revolution Square (35°58′N, 53°43′E) in Semnan, Iran in three different periods, namely, before, during, and after a COVID-19 lockdown.

### Data collection

Hourly records of individual pollutant concentrations of PM_2.5_, O_3_, NO_2_, and CO concentrations and AQI values from 1 March 2019 to 27 February 2021 were obtained from the website of the Iranian Air Pollution Monitoring System (https://aqms.doe.ir). The network sharing covers the online data and send/publish the information according to the technical guidelines of quality management of environmental monitoring. In total, 985 air pollution observations were collected, and were distributed over an average of 324 days for the city studied.

### Mathematical model

#### Air quality index

To investigate changes in air pollution, the US AQI index was used in this study. The US AQI is one of the best-known indicators for air quality communication (EPA 2018). This index converts values of pollutant concentrations to a color scale of 0–500 units, where higher values indicated by a color change from green to violet mean higher health risks related to inhalational exposure (35). The US AQI “good” range (<12 μg m^−3^) is slightly higher than the WHO air quality guideline (<10 μg m^−3^) (Table 2). AQI values were calculated based on air pollutants concentrations obtained from the air monitoring station in Semnan, Iran according to US Environmental Protection Agency (USEPA) guidelines (36). According to the USEPA standard, the overall AQI index value is the maximum AQI value among six pollutants included for analysis. Moreover, the pollutant with the highest AQI value is called the critical/dominant pollutant. The three different periods used in this study were 1 March to 27 February for each year from 2019 to 2021: before lockdown (BF), 1 March 2019 to 27 February 2019; during lockdown (LD) 1 March 2020 to 27 February 2020; and after lockdown (AF), 1 March 2021 to 27 February 2021.

**Table 2:** The US Environmental Protection Agency (USEPA) air quality index (AQI) categories and its breakpoints (USEPA 2018); PM_2.5_, particulate matter with an aerodynamic diameter of <2.5 μm.

**O_3_ 8 h (ppm)**	**PM_2.524h_ (μg m^−3^)**	**CO _8h_ (ppm)**	**NO_2_ 1h (ppm)**	**AQI value**	**Air quality categories**
0~0.059	0.4~15	0.4~4	0~0.053	0~50	Good
0.060~0.075	15.5~35	4.5~9.4	0.054~0.1	51~100	Medium
0.076~0.095	35.1~65.4	9.5~12.4	0.101~0.360	101~150	Unhealthy for sensitive groups
0.096~0.115	65.5~150.4	12.5~15.4	0.361~0.640	151~200	Unhealthy
0.116~0.374	150.5~250.4	15.5~30.4	0.65~1.24	201~300	Very unhealthy
0.405~0.604	250.5~500.4	30.5~50.4	1.25~2.04	301~500	Dangerous

Daily AQI data for four pollutants represent average values calculated based on measurements at the monitoring station. All measuring dates were based on Coordinated Universal Time (UTC). Daily air quality records were obtained from the website of the Iranian Air Pollution Monitoring System (https://aqms.doe.ir) and USEPA (37, 38). To calculate AQI values, Equation 1 was used (37):


(1) $$\begin{array}{l} I_{P}=\frac{\left(I_{Hi}-I_{Lo}\right)}{\left(BP_{Hi}-BP_{Lo}\right)}\left(C_{P}-BP_{Lo}\right)+I_{Lo}I_{P} \end{array}$$

where *I*_*P*_ is the AQI for pollutant (p), *C*_*P*_ is the concentration of pollutant p, *BP*_*Hi*_ is the concentration breakpoint that is ≥ *C*_*p*_, *BP*_*Lo*_ is the concentration breakpoint that is ≤ *C*_*p*_, *I*_*Hi*_ is the index breakpoint corresponding to *C*_*high*_, and *I*_*Lo*_ is the index breakpoint corresponding to *C*__*Lo*_w_.

### AirQ^+^ model

In this study, the AirQ^+^ model was used to quantify health consequences attributed to PM_2.5_. This software was developed and distributed by the WHO to estimate the short-term effects of exposure to air pollutants in a certain period on the health of residents of a region. Based on the number of deaths classified by age in Semnan, Iran during the years 2019–2021 obtained from the Ministry of Health, Treatment, and Medical Education, the baseline incidence (BI) rate of natural deaths (due to all natural causes) and other deaths and mortality from chronic obstructive pulmonary disease (COPD), ischemic heart disease (IHD), lung cancer (LC), and stroke were calculated using the integrated exposure–response (IER) function (Table 3).

**Table 3:** Relative risk (RR) indexes, baseline incidence, and at-risk population due to long-term exposure to PM_2.5_ in Semnan, Iran in 2019–2021.

**Pollutant**	**Health outcome**	**RR per 10 μg m^−3^ (95% CI)**	**At-risk population**			**Baseline incidence (per 100,000)**		
			**2019**~**2020**	**2020**~**2021**	**2021**~**2022**	**2019**~**2020**	**2020**~**2021**	**2021**~**2022**
**PM**_**2.5**_	Natural mortality	1.062 (1.04~1.083)	99,140	99,290	99,418	806.5	806.5	806.5
	Stroke mortality	IER function	83,101	83,101	83,101	34	34	34
	COPD mortality	1.09 (1.04~1.14)	99,140	99,290	99,418	20	20	20
	LC mortality	IER function	99,140	99,290	99,418	15.55	15.55	15.55
	IHD mortality	IER function	121,596	121,780	121,937	147.33	147.33	147.33

PM_2.5_, particulate matter with an aerodynamic diameter of <2.5 μm. IER, integrated exposure-response; COPD, chronic obstructive pulmonary disease; LC, lung cancer, IHD, ischemic heart disease.

AirQ^+^ estimates the attributable proportion, attributable cases per 100,000 population at risk, and the proportion of cases in a range of concentrations of air pollutants (baseline incidence of health effects, desired concentration cutoff value of, and relative risk [RR]). To estimate the health effects attributed to PM_2.5_, the following method was used: First, the linear-log concentration-response function was selected to calculate the relative risks of all causes of mortality (39). Chronic obstructive pulmonary disease (COPD) was calculated using evaluation and comparison between attributed ratios. With the log–linear function, high values for attributable death (AP) (about 20–30%) were obtained. The log–linear function for relative risk (RR) is as follows (Equation 2):


(2) $$\begin{array}{l} \text{RR}=e^{ß\left\{\left(1n\left(x+1\right)-1n\left(x_{0}-1\right)\right)\right\}} \end{array}$$

where *x* is the average annual concentration of *X*_0_, PM_2.5_ cutoff concentration for PM_2.5_ (10 μg m^−3^ for long-term effects and 25 μg m^−3^ for short-term effects in PM_2.5_ based on the annual and daily values of the WHO air quality guidelines), β is the risk coefficient resulting from a meta-analysis of epidemiological studies reported in AirQ^+^ software. The attributable proportion (AP), i.e., the percentage of mortality attributed to exposure to PM_2.5_ is calculated as follows (Equation 3):


(3) $$\begin{array}{l} \text{AP}=\left(\text{RR}-1\right)/\text{RR} \end{array}$$

Also, *P* is used to estimate the number of attributable mortality (*n*) in the target population, which is shown in Equation 4:


(4) $$\begin{array}{l} N=\text{AP}\times \text{BI}\times P \end{array}$$

where BI and *P* are the baseline mortality rates per 100,000 population and the number of population at risk, respectively. The relative risk values per 10 μg m^−3^ of PM_2.5_ (CI = 95%) for all mortality in long-term exposure to PM_2.5_ is presented in Table 2 (40). Integrated exposure–response function (IER) was used to estimate COPD, LC, IHD, and stroke mortality attributed to long-term exposure to a level higher than the air quality guidelines of the WHO (e.g., 10 μg m^−3^ PM_2.5_). IER function was obtained from studies of ambient air pollution, second-hand tobacco smoke, combustion of fossil fuel for cooking at home, and active smokers (41).

The IER function is calculated as follows:


(5) $$\begin{array}{l} \text{IER}=1+\alpha \left\{1-\text{exp}\left(\gamma {\left(x-x_{0}\right)}^{\delta }\right)\right\} \end{array}$$

where the parameters γ, α, and δ are estimated by non-linear regression methods and determine the general shape of the non-linear concentration–response relationship. Functions are preprogrammed into the AirQ^+^. For IHD and stroke, we used GBD 2013 (Integration Function 2015) for all age groups over 25 years. The counterfactual concentrations for these functions are <10 μg m^−3^, so we subtract the effects of contamination <10 μg m^−3^ from the estimated effects of actual concentrations. For COPD and lung cancer, we used global burden of disease (GBD) 2015/2016 (comprehensive function 2016 against the air quality guideline value of the WHO) for all age groups over 30 years.

## Results and discussion

**Figure 2:**
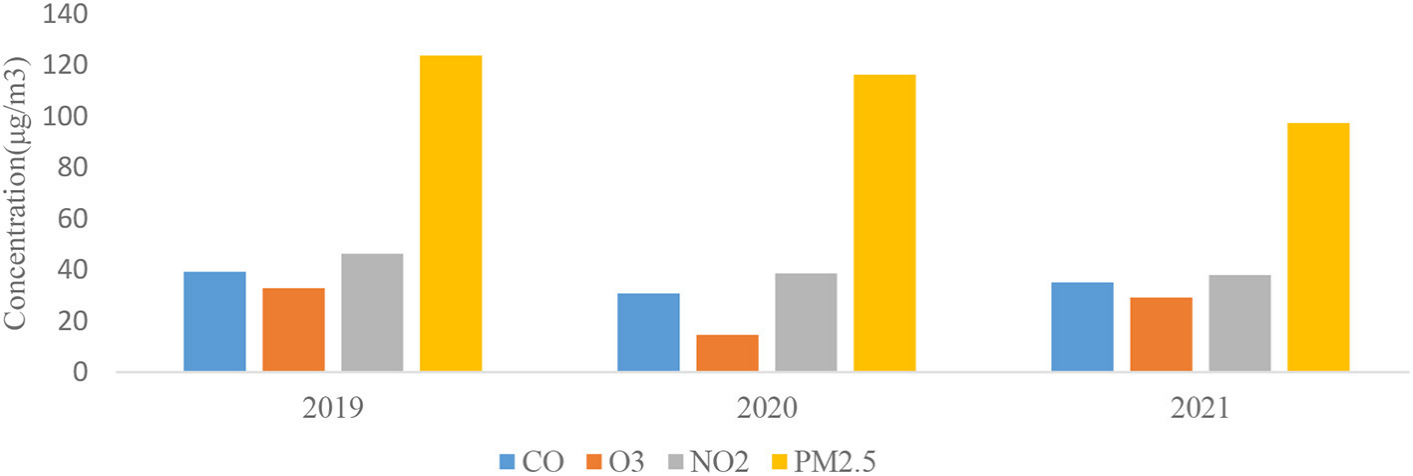
Annual variations of investigated air pollutants in Semnan Province, Iran in particular years from 2019 to 2021.

**Figure 3:**
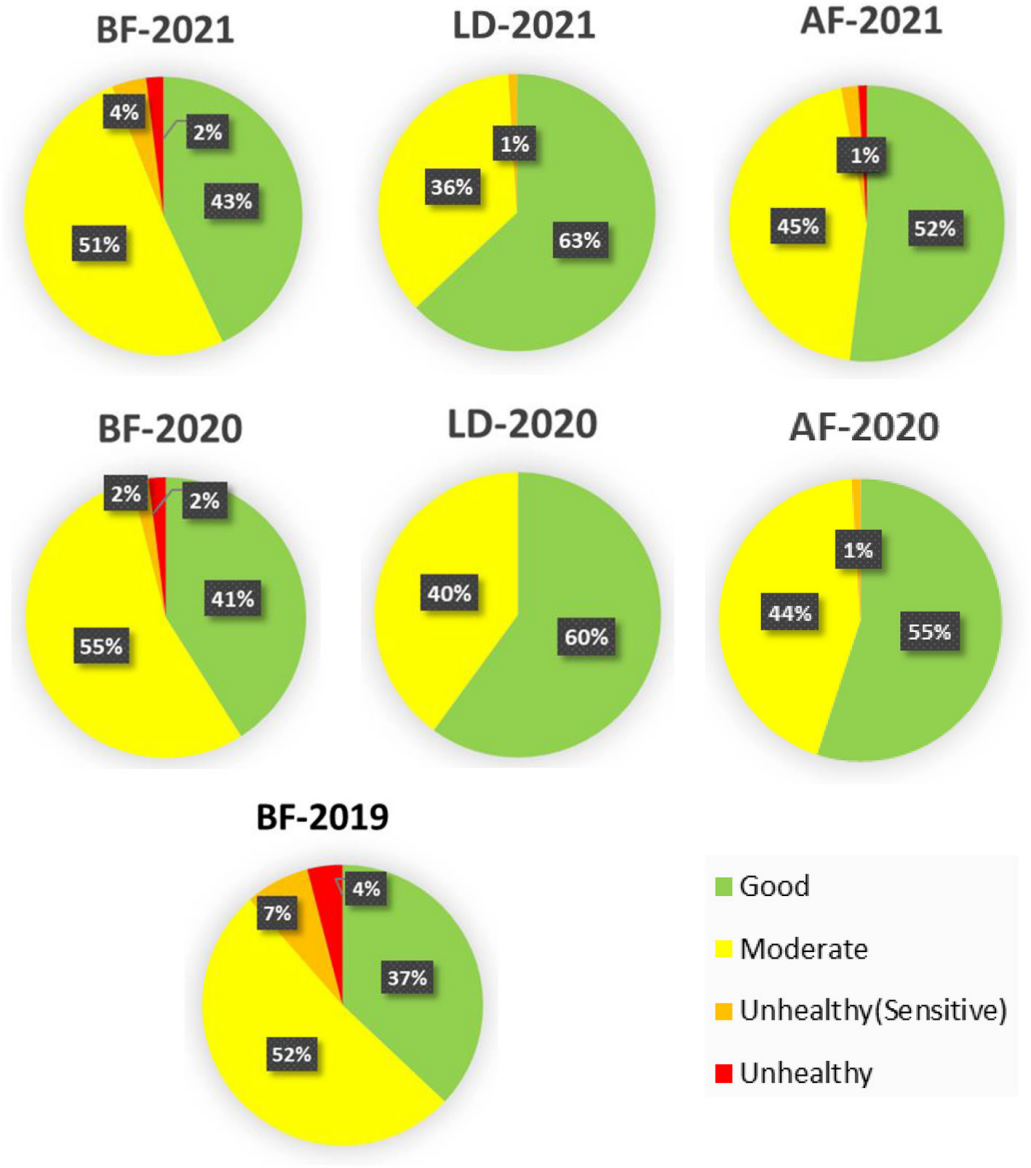
Overall distributions of air quality index (AQI) categories for before lockdown (BF), during lockdown (LD), and after lockdown (AF) in 3 years (2019–2021).

According to WHO guidelines from 2021 (10 μg m^−3^ for PM_2.5_ and 20 μg m^−3^ for NO_2_, annual average) recommended maximum concentrations were exceeded 19.8 times for PM_2.5_ and 1.8 times for NO_2_, respectively. The annual average concentrations of NO_2_ and PM_2.5_ do not exceed Canadian Ambient Air Quality Standards (CAAQS) Grade II standard levels (40 μg m^−3^ for NO_2_, and 35 μg m^−3^ for PM_2.5_, annual average) in all three study periods. Co and O_3_ do not have annual standards under CAAQS; CO and O_3_ decreased in Semnan, Iran during the lockdown (2020) compared with before lockdown (2019), and after lockdown (2021) (Figure 2). The AQI index informs the public on air quality so that individuals and communities can take adequate measures to protect their health. This measurement is especially critical on days with unhealthy air quality (AQI >100). Based on the results, the dynamic degree related to air pollution in the lockdown and postlockdown periods depended on the pollution level in the area. The percentages of the overall AQI categories in the three analyzed periods of before (BF), during (LD), and after (AF) lockdown in 2019, 2020, and 2021 indicated that the percentages of days with good air quality were the highest during lockdown periods: 60% in 2020 and 63% in 2021 of the days during the year (Figure 3). After lockdowns, the percentage of the days with good AQI air quality category (AQI <50) decreased (52% in 2020 and 55% in 2021) at the expense of increases in the days with the moderate AQI air quality category from 40 to 44% in 2020 and from 36 to 45% in 2021. Regarding the unhealthy air quality category determined in Semnan, Iran, it was revealed that the highest shares of days for this category before the lockdown were 4% in 2019, 2% in 2020, and 2% in 2021. After the lockdown, the unhealthy air quality category did not appear in 2020 or 2021, covering 1% of the share. Regarding the unhealthy category of air pollution for sensitive subpopulations, a similar trend was observed: the highest share among air quality categories was before the lockdown (7% in 2019, 2% in 2020, and 4% in 2021). The percentage of days in the unhealthy air pollution category for sensitive groups returned to 1% after the lockdown and then remained at the same level for the same period in 2020. Therefore, the number of days in the unhealthy air quality category (AQI >100) decreased during the lockdown and returned to the average level after the lockdown was lifted.

**Figure 4:**
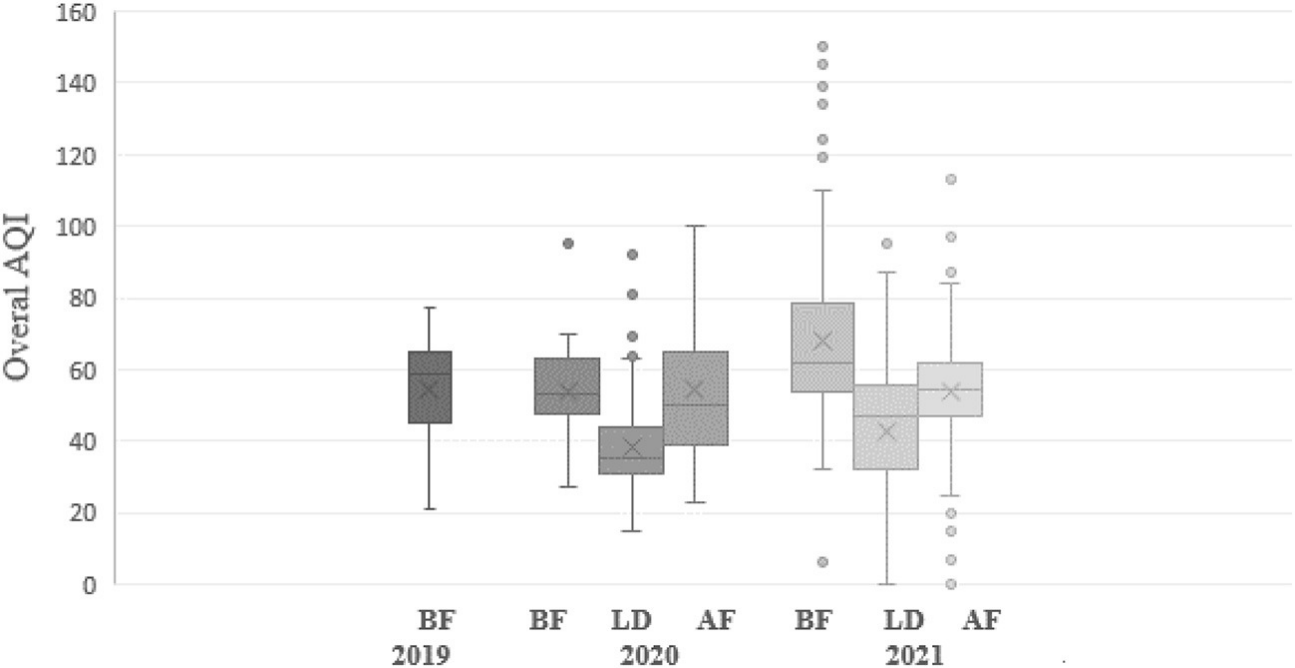
Average daily overall air quality index (AQI) values in Semnan, Iran for the study periods: before lockdown (BF), during lockdown (LD), and after lockdown (AF) in 2019–2021.

The general trend of the overall AQI values is shown in Figure 4. The AQI index was the lowest during lockdown periods. In 2020, it was observed that after the lockdown period, the level of air quality remained lower than before lockdown in 2020. In 2021, the air pollution level was even higher than before lockdown. This could have been due to the still uncertain situation regarding the COVID-19 pandemic in 2020, while in 2021, recovery from the pandemic was more certain. This affected to a large extent the return to normal activities including anthropogenic emissions from traffic and industry. In 2020, when the pandemic was still uncertain, governmental actions limiting various human activities lowered air pollution as a side effect.

### Evaluation of air pollution using the overall AQI

Regarding the dominant role of individual pollutants in total AQI values and pollution categories during the 3-year study period, it was revealed that among the four investigated pollutants, PM_2.5_ for each period in each year was the pollutant with the highest sub-AQI (AQIi) values (Table 4).

**Table 4:** Average sub-air quality index (AQIi) values for air pollutants in Semnan, Iran for the study periods: before lockdown (BF), during lockdown (LD), and after lockdown (AF) in 2019–2021.

	**AQI values**						
	**2019**	**2020**			**2021**		
**Pollutant**	**BF**	**BF**	**LD**	**AF**	**BF**	**LD**	**AF**
CO	37.3	33.3	31.4	27.4	50.1	33.9	31.2
NO_2_	49.9	47.8	24.5	27.6	43.2	31.8	36.4
O_3_	37.9	10.4	22.7	14.7	56.3	30.0	29.5
PM_2.5_	109.0	70.1	39.9	40.5	55.9	43.1	51.8

PM_2.5_, particulate matter with an aerodynamic diameter of <2.5 μm.

To determine the critical pollutant in the overall AQI and to estimate the contributions of the four pollutants evaluated in this study, the average values for each day from before (BF), during (LD), and after (AF) the lockdown periods were calculated and classified according to AQI categories, as defined by the USEPA (Table 2). If the AQI value for an individual pollutant exceeded 100, this pollutant was considered to be a critical pollutant. Our results showed that the average AQIi value for PM_2.5_ exceeded 50, while the AQIi values for CO, NO_2_, and O_3_ were below 50 in all investigated periods (Table 4). The AQIi value of PM_2.5_ was the highest among individual AQIi values of the other pollutants. Based on this, the overall AQI was determined based on Equation (1). It was shown that PM_2.5_ was a critical pollutant (Table 5) and the primary pollutant responsible for the unhealthy category of air quality on most days. PM_2.5_ is primarily generated by physical processes, including soil particle resuspension, road dust, sea spray, agricultural tillage, and transportation and industrial activities. These sources of PM_2.5_ emissions significantly decreased during the lockdown period in 2020–2021. Considering that PM_2.5_ was a critical pollutant in subsequent parts of this research, the mortality attributed to this pollutant was calculated. The average AQIi values for NO_2_ during the prelockdown period in the 3 years of the study were 49.9, 47.8, and 43.2, which were <50, and therefore the air quality was classified as good (Table 4). The average AQI values of NO_2_ for the BF, LD, and AF periods showed a decreasing trend compared to 2019. For Semnan, Iran, the AQI values for NO_2_ decreased during LD periods compared to the same period in 2019 (Table 4). However, the AQI for NO_2_ returned to the level before the pandemic after the end of the lockdown in Semnan, Iran. Thus, there was a significant reduction in the AQIi for NO_2_ during the short-term lockdown. However, no significant changes were observed after the lockdown period in the final 2 years. In cities, fossil fuel combustion is often the primary source of air pollutants and includes stationary electricity generation, and diesel and gasoline engines. There is some concern that diesel after-treatment technologies aimed at reducing PM emissions could shift the distribution of NO_x_ emissions toward NO_2_, leading to greater exposure to NO_2_ near highways. The average AQIi values for O_3_ in Semnan, Iran for the prelockdown period of the study were 37.9, 10.4, and 56.3, indicating good air quality (Table 4). The average AQI values for O_3_ for the BF, LD, and AF periods showed an increasing trend for the 3 years. In addition, the AQIi for O_3_ significantly increased after the end of the lockdown period in 2021 compared to the same period in 2020. The average AQIi values for CO in the studied years were between 27.4 and 51.6, and thus below 100 (Table 4). The average AQIi value for CO significantly decreased in 2020. Mean AQIi values for PM_2.5_ were very high compared to the other three air pollutants and ranged from 39.9 to 78.6. Therefore, according to the AQI categories, the air quality for these values was classified as moderate (Table 3). PM_2.5_ emissions from natural sources are usually much higher than from anthropogenic emissions. The average AQIi values for PM_2.5_ before lockdown (1 March 2019 to 27 February 2019) indicated that the air quality category was unhealthy for sensitive groups with a mean value of 109.0 μg m^−3^. However, during the national COVID-19 lockdown during 2020–2021, the AQIi values decreased, and the air quality was classified as good with a PM_2.5_ concentration range of 39.9 to 43.1 μg m^−3^. Due to the full implementation of the national lockdown from March 2020 to the last week of August 2020, PM_2.5_ concentrations decreased. According to Table 5, concentrations of most pollutants decreased in 2021, but in different proportions, including CO (a 15% decrease), O_3_ (a 3% decrease), and NO_2_ (a 26% decrease).

**Table 5:** Contributions (%) of individual pollutants to average Air Quality Index (AQI) values in Semnan, Iran from 1 March to February 27 in 2019–2021.

**Pollutant**	**2019**	**2020**	**2021**
CO	17%	15%	5%
NO_2_	21%	26%	16%
O_3_	8%	3%	1%
PM_2.5_	54%	45%	78%

PM_2.5_, particulate matter with an aerodynamic diameter of <2.5 μm.

### Quantifying health impacts of PM_2.5_ in Semnan, Iran in 2019–2021

**Figure 5:**
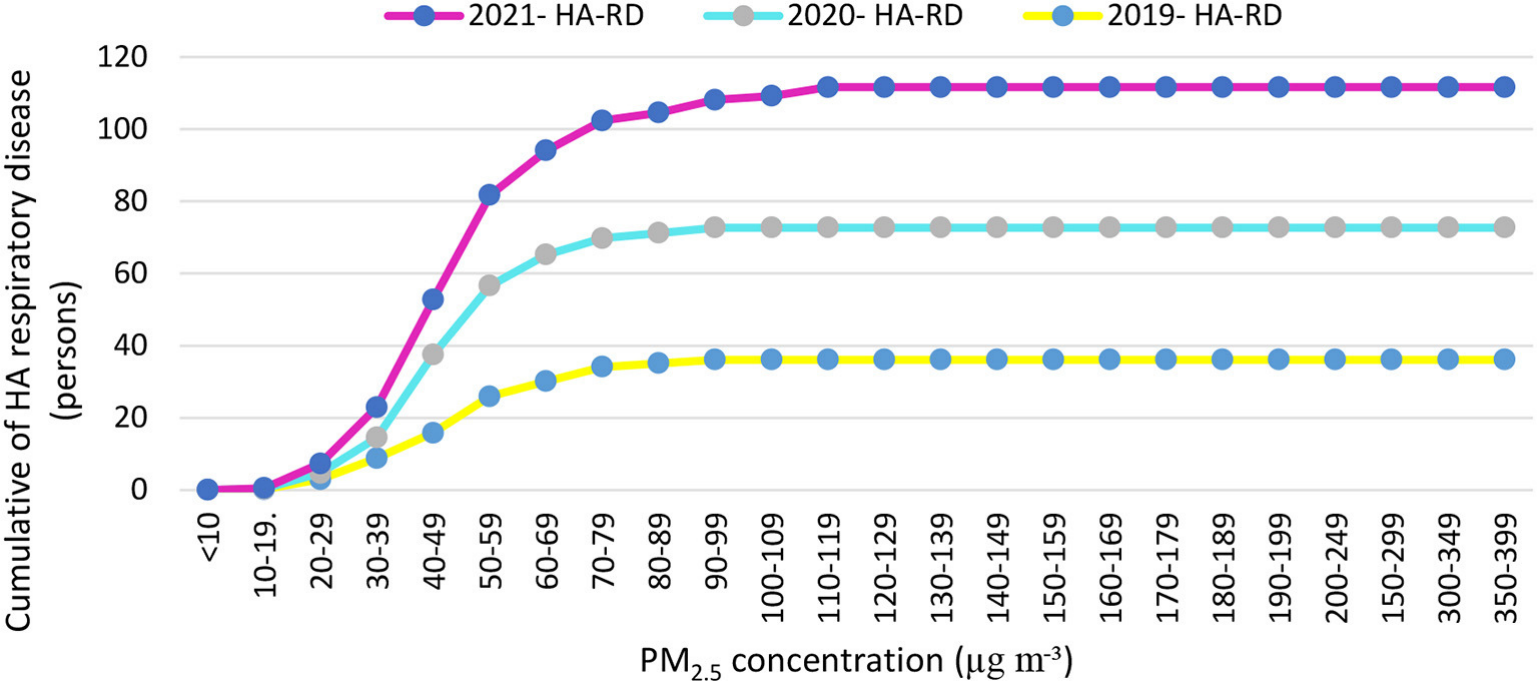
Cumulative numbers of cases of the hospital admission (HA) referrals due to respiratory diseases attributed to particulate matter with an aerodynamic diameter of <2.5 μm (PM_2.5_) in concentration intervals in Semnan, Iran in 2019–2021.

**Figure 6:**
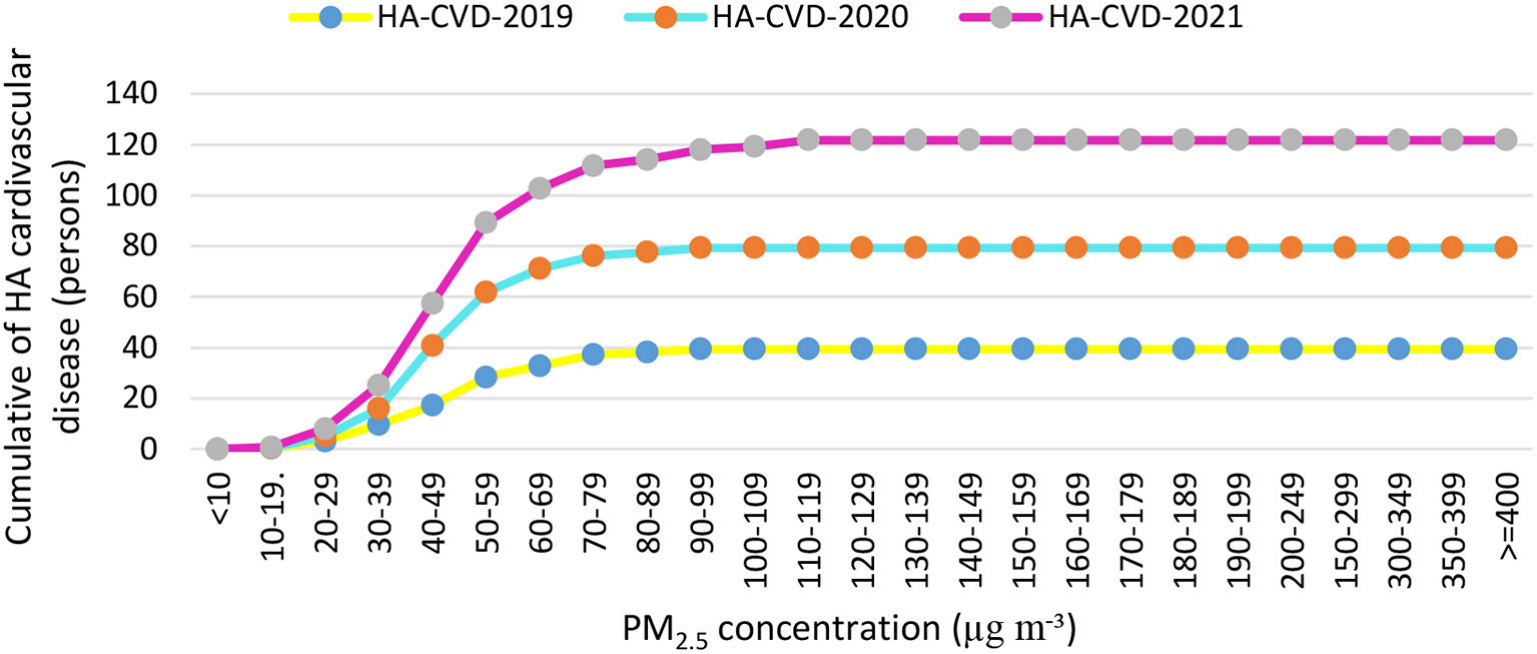
Cumulative numbers of cases of the hospital admission (HA) referrals due to cardiovascular diseases attributed to particulate matter with an aerodynamic diameter of <2.5 μm (PM_2.5_) in concentration intervals in Semnan, Iran in 2019–2021.

This study determined the basis of quantifying mortality and morbidity using the AirQ^+^ model. PM_2.5_ concentrations are presented in Table 3 according to recorded concentrations of the Semnan, Iran air pollution monitoring station in 2019, 2020, and 2021. The estimated relative risk (RR) indices of the attributed component and additional deaths attributed to PM_2.5_ are described in Tables 3, 6 and Figure 4. The average concentration of PM_2.5_ ranged from 97.30 to 123.34 μg m^−3^. The maximum annual concentration of PM_2.5_ was recorded with an average range of 102.18 to 148.87 μg m^−3^ from 2019 to 2021 (Table 6). In the present study, based on RR indices and the baseline incidence (BI) listed in Table 3, the number of hospital admission (HA) cases and deaths due to cardiovascular and respiratory diseases attributed to the effects of PM_2.5_ in three low RR indices (5% RR), central and high (95% RR) were estimated in Table 7, and Figures 5, 6. Figures 5, 6 show that the number of hospital admissions attributed to PM_2.5_ in Semnan, Iran decreased during the national lockdown period. Therefore, cumulative mortality totals from COPD, IHD, LC, and stroke attributed to PM_2.5_ during 2019~2021 were calculated. Based on the results in Table 6, 2019 had the highest number of mortalities, and 2021 had the minimum mortality numbers attributed to criteria air pollutants in the study period. Based on the results in Table 6, 2019 had the highest mortality levels, and 2020 had the lowest mortality levels from COPD, IHD, LC, and stroke attributed to PM_2.5_ in the study period. Results in Table 3 show that considering a BI of natural mortality of 806.5 per 100,000 people, the cumulative frequency of this consequence in 2021 was 202 people, which had decreased by 27 people compared to 2019. In 2019, the highest number of hospital admissions for cardiovascular disease (120 people) was related to concentrations higher than 100 to 110 μg m^−3^. Based on Figure 5, the cumulative frequency of respiratory diseases attributed to PM_2.5_ was estimated to be 110 people in 2019, which had decreased by 39 people compared to 2021.

**Table 6:** Particulate matter with an aerodynamic diameter of < 2.5 μm (PM_2.5_) concentrations (μg m^−3^) in Semnan, Iran in 2019–2021.

**Parameter**	**Year**		
	**2019**	**2020**	**2021**
	μ**g m**^−3^		
Annual mean	123.87	116.30	97.34
Winter mean	95.23	87.21	58.94
Summer mean	112.35	105.51	98.47
Annual 98^th^ percentile	123.56	111.5	98.32
Summer maximum	145.76	116.23	102.45
Winter maximum	148.65	123.18	117.87

**Table 7:** Attributable proportions and cases due to long-term exposure to particulate matter with an aerodynamic diameter of <2.5 μm (PM_2.5_) in 2019–2021.

**Pollutant**	**Health outcome**	**Attributable proportion (%)**			**Attributable cases**		

		**2019**~**2020**	**2020**~**2021**	**2021**~**2022**	**2019**~**2020**	**2020**~**2021**	**2021**~**2022**
PM_2.5_	Natural mortality	28.6 (19.72~36.01)	24.17 (16.51~30.7)	23.49 (16.02~29.87)	229 (158~289)	202 (138~257)	194 (132~246)
	Stroke mortality	23.46 (14.44~37.55)	21.2 (12.76~33.71)	20.83 (12.49~33.25)	7 (4~11)	6 (4~9)	6 (4~10)
	COPD mortality	25.18 (16.57~37.93)	22.55 (14.64~33.85)	22.12 (14.33~33.13)	5 (3~8)	4 (3~7)	4 (3~7)
	LC mortality	23 (16.03~28.96)	20.22 (13.82~25.85)	19.77 (13.46~25.33)	4 (2~4)	3 (2~4)	3 (2~4)
	IHD mortality	25.4 (17.58~42.29)	23.26 (15.86~43.96)	22.91 (14.58~43.41)	45 (31~85)	41 (28~78)	42 (28~79)

COPD, chronic obstructive pulmonary disease; LC, lung cancer; IHD, ischemic heart disease.

#### The relationship between lockdowns and air pollutants

Air pollution has also been called a “silent global health disaster,” an outbreak that kills seven million people annually, making it a more serious threat than any other form of exposure (42, 43). Many studies showed that in addition to reducing and controlling the COVID-19 epidemic, national lockdown actions led to improved air quality (44–47). Based on the results of this study, the main pollutant in the air of Semnan, Iran was identified as PM_2.5_. Annual concentrations of PM_2.5_ in the years before, during, and after COVID-19 in Semnan, Iran were, determined to be 123.9, 116.3, and 97.3 μg m^−3^, respectively, which were 3.51, 3.31, and 2.78 times higher than those presented in WHO guidelines. One of the reasons for the increase in the concentration of suspended particles in the ambient air in cities is dust storms that enter Iran from the western and southwestern regions in recent years. Until recently, these dust storms were only seen in spring and summer, but nowadays, this phenomenon can be seen in most months of the year, which affects most of the regions of Iran, especially the southwestern regions. Based on the results of this study, the average AQIi values of PM_2.5_ during and after the lockdown period in 2020 were 39.9 and 40.5 μg m^−3^ and in 2021 were 43.1 and 51.8 μg m^−3^, respectively. Average concentrations of criteria air pollutants during lockdowns were lower than both National Ambient Air Quality Standards (NAAQS) (48) and WHO guidelines (49). This could have been due to reductions in the activities of transportation facilities and industrial processes during those years. Similar results were found for criteria pollutants which were 2–3 times higher than WHO guidelines in the years before and after the lockdowns (50). In this study, a significant improvement in AQI values was observed during the lockdown period, especially for PM_2.5_ from a maximum value of 109.0 to a minimum value of 39.9, and for NO_2_ from a maximum value of 49.9 to a minimum value of 24.5. This improvement was due to the reduced greenhouse gas emissions in the transportation and industry sectors. Before the lockdown period, the AQI indicated generally poor to moderate air quality categories in six industrial cities (Tehran, Tabriz, Mashhad, Urmia, Ahvaz, and Arak) (51). In contrast, during the COVID-19 lockdown period, the AQI indicated good air quality categories on average, ranging from satisfactory to moderate categories. Our study detected a 35.1% decrease in the AQI value during the COVID-19 lockdown period compared to before the lockdown. Approximate increases of 19.6% and 11.1% were observed in AQI values between the COVID-19 lockdown and after. Therefore, the high AQI values during the implementation of government intervention measures might have been mainly influenced by heavy pollution from industrial sources. The AQI values showed significant temporal differences due to industrial greenhouse gas emissions in Semnan, Iran where the COVID-19-related lockdown prevented those emissions. It was reported that reducing emissions of suspended particles had the greatest effect on improving air quality during the lockdown (44), but this pollutant increased after the end of the lockdown. Some studies showed that PM_2.5_ can cause the spread of COVID-19 and increase mortality (52, 53). Those results imply that greater control of regional transport activities is a key factor in reducing pollutant levels, because regional transport was severely restricted during the lockdown, and there were restrictions on human activities, transportation, and factories during the lockdown period. In this study, with an increase of 10 μg m^−3^ in PM_2.5_ concentrations, the risk of attributed respiratory diseases in Semnan, Iran increased by 0.8%. A systematic review of the prediction of health effects in Iran showed that levels of most air pollutants were higher than presented in WHO guidelines in 2021 and were predicted to lead to significant adverse health effects in different cities of Iran (54). In Ahvaz, Iran, from 2014 to 2018, the annual average PM_2.5_ was 5.2–8 times higher than air quality guidelines (10 μg m^−3^). PM_2.5_ caused the average ages of the total population, people aged between 0 and 64 years, and people over 65 years to decrease by 2.5, 3, and 1.6 years, respectively (55). From the point of view of public health and health risks, PM is one of the main threats to human health, especially in large cities where air pollution levels exceed daily limits (56). It is estimated that 500,000 people die annually from exposure to PM worldwide (57). The low PM_2.5_ concentrations were associated with high cumulative hospital admissions during 2019–2021. During a lockdown, Burnett et al. (58) conducted a preliminary analysis of PM_10_ and possible human hospital admissions in Toronto, Canada, and they reported that exposure to PM_10_ could have caused a 40.4% increase in civilian hospital admissions. According to the results of this study, ~1.4% of hospital admissions occurred when the PM_10_ concentration was higher than 20 μg m^−3^ (58). The hospital admission rates in the Iranian cities of Ahvaz, Bushehr, and Kermanshah were estimated to be 1.5, 2.7, and 1.9%, respectively, when the PM_10_ concentration was above 20 μg m^−3^ (59). In another study of six Italian cities, there was a significant relationship between SO_2_ concentrations and health effects on inhabitants (60). It was also proven that PM_2.5_ seriously affects health and increases deaths caused by respiratory and cardiovascular diseases, and lung cancer (61). With long-term exposure, every 10 μg m^−3^ increments in PM_2.5_ increases the mortality rate by 6%, previous vascular diseases by 12%, and lung cancer by 14% (62, 63). One study also showed that dust storms caused a 1.7% increase in deaths (64). In this study, the hospital admissions of residents of Semnan, Iran due to exposure to PM_2.5_ were evaluated using the AirQ^+^ model. Based on the results of our study, the greatest cumulative numbers of hospital admissions due to cardiovascular diseases attributed to PM_2.5_ in 2019, 2020, and 2021 were 27, 33, and 28 cases, respectively. The annual concentration of PM_2.5_ (ca. 31 μg m^−3^) did not change significantly from 2016 to 2018 in Tehran and was almost 3 times higher than that presented in WHO guidelines (65). Premature deaths from long-term exposure to PM_2.5_ in Turkey using the AirQ^+^ program showed that 44,617 people (95% confidence interval: 29.882~57.709) died prematurely in 2018. The highest estimated number of deaths attributed to PM_2.5_ pollution was in Manisa and Afyon Karahisar Provinces (66). Investigating the health effects of PM_2.5_ using the AirQ^+^ model in Semnan, Iran, showed that 4% of all respiratory deaths were related to concentrations of >20 μg m^−3^ (67). Results of the measurement of particles in the air of Semnan, Iran showed that the annual average concentrations of PM_2.5_ decreased from 2019 to 2021, which reached 97.3 μg m^−3^ in 2021. The maximum annual average concentration of PM_2.5_ was 148.65 μg m^−3^, observed in the winter of 2019. The results obtained from the compilation of epidemiological indicators, considering the average relative risk in Semnan, Iran showed that mortality rates from COPD attributed to PM_2.5_ in 2019–2021 were 25.18, 22.55, and 22.12%, respectively. Considering the baseline incidence of 427 people per 100,000 hospital admission caused by cardiovascular diseases, cumulative numbers in 2019–2021 were 612, 898, and 924, respectively. In summary, 37% of all deaths in 2019, 38% in 2020, and 43% in 2021 occurred on days with concentrations lower than 400 μg m^−3^. Hospital admissions related to respiratory diseases in 2019–2021 were 27.2, 25.6, and 19.6%, respectively. Based on the results of this study, the effectiveness of lockdowns on pollution in Semnan, Iran was evident, and this shows the vigilance and obedience demonstrated by the people.

## Conclusion

This study comprehensively evaluated the air quality in Semnan, Iran related to the COVID-19 pandemic based on three different time periods, namely, before the lockdown (BF) from 1 March 2019 to 27 February 2019, during the lockdown (LD) from 1 March 2020 to 27 February 2020, and after the lockdown (AF) from 1 March 2021 to 27 February 2021, based on the AQI and AirQ^+^ models. PM_2.5_ was found to be a critical/dominant pollutant with contributions to total AQI values of 54% in 2019, 45% in 2020, and 78% in 2021. The results in this study indicated that good air quality was observed during lockdown periods in 60% of the days in the year 2020 and 63% of the days in 2021. Regarding the AQI values for particular pollutants, significant decreases were observed during the lockdown period in our study for PM_2.5_ from a maximum value of 109.0 to a minimum value of 39.9 and for NO_2_ from a maximum value of 49.9 to a minimum value of 24.5. An unhealthy air quality category was observed before the lockdown on 4% of days in 2019 and 2% of days in 2020 and 2021. After the lockdown, the unhealthy air quality category was not observed in 2020 and on 1% of days in 2021. However, after the lockdown, the AQI revealed unhealthy air quality for susceptible subpopulations. The RR indices and the BI revealed that the number of hospital admissions due to PM_2.5_ pollution in Semnan, Iran decreased during the national lockdown period. The highest mortality rates attributed to air pollution and due to COPD, IHD, LC, and stroke diseases were revealed in 2019, while the lowest mortality rates from these causes in 2021 with the minimum mortality were attributed to criteria air pollutants in the study period. Based on results in Table 5, 2019 had the highest mortality, and 2020 had the lowest mortality from COPD, IHD, LC, and stroke attributed to PM_2.5_ in the study period. Our results support the general finding that anthropogenic activities cause significant health threats, as paradoxically revealed during a global health crisis/challenge.

## Data availability statement

The raw data supporting the conclusions of this article will be made available by the authors, without undue reservation.

## Ethics statement

The Research Ethics Committee of Kashan University of Medical Sciences (KAUMS) granted ethics approval for this study (No. IR.KAUMS.NUHEPM.REC.1401.0).

## Author contributions

Conceptualization, methodology, project administration, and resources: SG, AK, GM, AG-K, and PH. Data curation, Formal analysis, and writing—original draft: SG and AK. Investigation: SG, AK, GM, AG-K, K-JC, and PH. Software: SG. Writing—review and editing: AK, GM, AG-K, K-JC, and PH. All authors contributed to the article and approved the submitted version.
